# Tissue-Specific Analysis of Secondary Metabolites Creates a Reliable Morphological Criterion for Quality Grading of Polygoni Multiflori Radix

**DOI:** 10.3390/molecules23051115

**Published:** 2018-05-08

**Authors:** Li Liang, Jun Xu, Zhi-Tao Liang, Xiao-Ping Dong, Hu-Biao Chen, Zhong-Zhen Zhao

**Affiliations:** School of Chinese Medicine, Hong Kong Baptist University, Hong Kong, China; liangrb.ll@gmail.com (L.L.); davidxujun@hkbu.edu.hk (J.X.); liangzhitao23@gmail.com (Z.-T.L.); dongxiaoping11@126.com (X.-P.D.); hbchen@hkbu.edu.hk (H.-B.C.)

**Keywords:** Polygoni Multiflori Radix, laser microdissection, ultra-performance liquid chromatography triple-quadrupole mass spectrometry (UPLC-QqQ-MS/MS), secondary metabolites, quality grading

## Abstract

In commercial herbal markets, Polygoni Multiflori Radix (PMR, the tuberous roots of *Polygonum multiflorum* Thunb.), a commonly-used Chinese medicinal material, is divided into different grades based on morphological features of size and weight. While more weight and larger size command a higher price, there is no scientific data confirming that the more expensive roots are in fact of better quality. To assess the inherent quality of various grades and of various tissues in PMR and to find reliable morphological indicators of quality, a method combining laser microdissection (LMD) and ultra-performance liquid chromatography triple-quadrupole mass spectrometry (UPLC-QqQ-MS/MS) was applied. Twelve major chemical components were quantitatively determined in both whole material and different tissues of PMR. Determination of the whole material revealed that traditional commercial grades based on size and weight of PRM did not correspond to any significant differences in chemical content. Instead, tissue-specific analysis indicated that the morphological features could be linked with quality in a new way. That is, PMR with broader cork and phloem, as seen in a transverse section, were typically of better quality as these parts are where the bioactive components accumulate. The tissue-specific analysis of secondary metabolites creates a reliable morphological criterion for quality grading of PMR.

## 1. Introduction

Various specifications or grades of Chinese medicinal materials (CMMs) can be found in the market. The upper grade CMMs are supposed to be of better quality, thus justifying higher prices. Today, for determining grade and price, medicinal vendors mostly use morphological features, both external and internal (e.g., stem cross-sections), including shape, size (diameter, length), color, and texture [1]. Similarly, consumers primarily evaluate the quality of CMMs on the basis of morphological features in herbal markets. For each CMM, the particular characteristics used for grading were fixed centuries ago. However, modern research has found that the traditional interpretations of morphological features used for commercial grading of CMMs do not always reliably correspond to quality [2,3]. The accurate correspondence between quality and grading/price is an important issue both for the commercial markets and for clinical use of CMMs.

Today, chemical analysis has been applied to determine the content of active components in different commercial specifications and grades of herbal medicine. Until recently, in most of the research, an entire CMM or a whole plant was taken as the basis of research [4,5,6]. Today, with advanced technology, histochemical studies can extend to tissue-level analysis, and have shown that different tissues of CMMs contain different and distinct types of secondary metabolites [7,8,9]. At the gross, macroscopic level, the structure of different tissues creates morphological features, which is the external form of the CMMs. Thus the relationship between bioactive components and morphological features can be found by analyzing the distribution patterns of chemical components in different tissues. As a result, tissue- and cell-specific chemical profiling can link morphological features with secondary metabolites [10,11,12]. As such, both entire CMM sample and tissue-specific chemical profiling can be used to link morphological features and the quality of CMMs.

Recently, the technique of ultra-performance liquid chromatography triple-quadrupole mass spectrometry (UPLC-QqQ-MS/MS) has been used for precise quantitative analyses of the complex mixture of multi-components in CMMs with superior sensitivity and specificity [13,14,15]. In particular, multiple reaction monitoring (MRM) is a powerful quantitative mode operated on QqQ-MS/MS, in which two quadrupole mass analyzers are comprised and programmed at selective scanning, allowing only one ion pair (precursor and product ions) to be detected. Since two stages of mass selectivity are utilized, there is little interference from the background matrix [16,17]. In addition, laser microdissection (LMD) has been used as an accurate sampling tool for precise, contamination-free preparation of cell groups or single cells excised from histological tissue sections without adjoining tissues adhering. In addition, an accurate cutting area can be recorded, with the degree of preciseness up to 1 μm^2^. [18,19]. A fluorescence light built inside the LMD unit enables each tissue or cell to appear with a specific fluorescence color according to its chemical constituents. This makes the border of each tissue more clear, enabling tissue or cell separation to be more accurate and specific [20]. The application of LMD and the UPLC-QqQ-MS/MS technique is proving to be a valuable technical platform for tissue- and cell-specific metabolite profiling of CMMs [21]. The method combining LMD and UPLC-QqQ-MS/MS can not only quantitatively analyze bioactive components of CMMs, but can also map the distribution patterns of bioactive components. Linking chemicals and morphological features of CMMs provides the scientific basis for their quality evaluation.

Polygoni Multiflori Radix (PMR, *heshouwu* in Chinese; derived from the tuberous roots of *Polygonum multiflorum* Thunb.) is widely used in traditional Chinese medicine. It is used to resolve toxin, moisten the intestines, and frees the stool [22]. Previous chemical analysis of PMR have found that it contains mainly three types of bioactive chemical ingredients: diphenyl ethylene, anthraquinones and polyphenols [23]. According to the *Compendium of Materia Medica* (*Ben Cao Gang Mu*), an ancient Chinese reference text, PMRs of a larger size are of better quality [24]. In the modern herbal market, based on our preliminary investigation in a local commercial herbal market, we found that PMR is sold in three grades according to their weight and size, with the price varying from 30 HKD (US$4) per kg for smaller pieces to 150 HKD (US$20) per kg for the largest pieces. Wholesalers grade PMR according to weight: grade 1 represents tuberous roots more than 100 g in weight; grade 2, between 50 g to 100 g in weight; and grade 3, below 50 g in weight [25]. Commercial herb dealers chop the herb into smaller pieces and grade/sell it accordingly. While both sellers and buyers accept this grading, two questions arise: does the existing grading system accurately represent the quality of the herb? If not, is there another system, or are there other morphological criteria, that could be used to reliably represent quality?

We performed preliminary qualitative histochemical analysis of PMR by means of fluorescence microscopy and high performance liquid chromatography time-of-flight mass spectrometry (HPLC-TOF-MS). However, due to the limitations of tissue-separating technology at that time, the tissue parts were only roughly separated manually [26]. Because some types of specific tissues or cells of PMR are tiny, with a thickness even in units of μm, and close together, they can be impossible to see and thus easily mis-cut manually; furthermore, the size of the cutting area is hard to measure, again due to its small size, and thus it is too inaccurate for quantitative study. Therefore, the analytes in the previous study were only qualitatively investigated without quantitative determination, which thus cannot reveal the histological distribution of chemicals in PMR.

**Figure 1:**
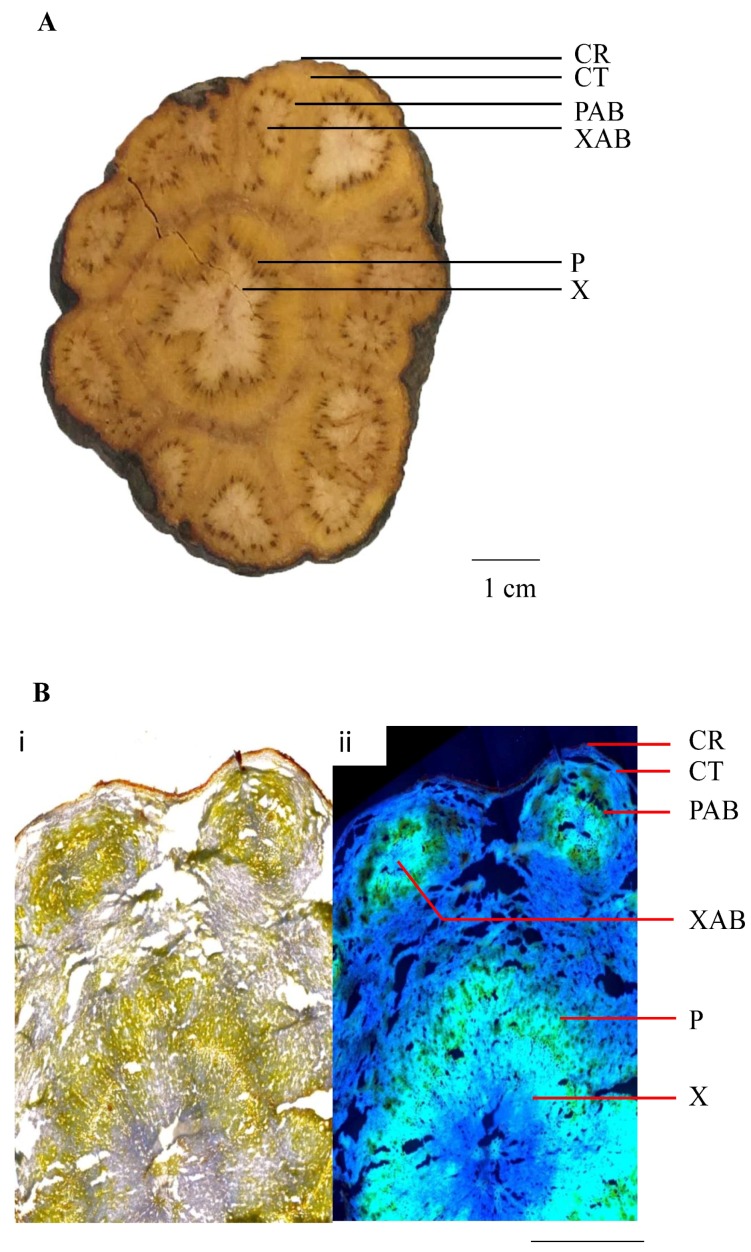
Transverse section of Polygoni Multiflori Radix (PMR) (PMR-TA1). (**A**) morphological features; (**B**) under microscope (6.3×). (i) Under normal light microscope; (ii) under fluorescence mode. CR: cork; CT: cortex; PAB: phloem of abnormal vascular bundles; XAB: xylem of abnormal vascular bundles; P: phloem; X: xylem.

In this study, we conducted a quantitative, tissue-specific histochemical analysis of PMR by combining LMD and UPLC-QqQ-MS/MS. The objective of this study is to determine whether and how morphology corresponds to quality. To achieve this goal, we firstly determined the contents of 12 major chemicals in three different commercial grades of PMR raw materials by UPLC-QqQ-MS/MS to explore whether the existing grading system of PMR accurately represent the quality of the herb. The 12 analytes were *trans*-2,3,5,4’-tetrahydroxystilbene-2-*O*-β-D-glucopyranoside (*trans*-THSG), *cis*-THSG, emodin, physcion, emodin-8-*O*-β-D-glucosides and physcion-8-*O*-β-D-glucosides, gallic acid, proanthocyanidin B1, proanthocyanidin B2, epicatechin, catechin and epicatechin-3-gallate; these have been widely reported to be the major types and major bioactive chemical components in PMR [26,27,28,29] and thus were used as evidence of quality for this purpose. Then, we used a combination method of LMD and UPLC-QqQ-MS/MS to map the distribution patterns of these 12 analytes in six different PMR tissues, namely cork, cortex, phloem of abnormal vascular bundles, xylem of abnormal vascular bundles, phloem and xylem (Figure 1), in order to find out other morphological criteria for the quality grading of PMR. The results provide the chemical basis for quality evaluation of PMR, and expand the evaluation criteria to include internal morphological features.

## 2. Results and Discussion

### 2.1. Sample Extraction

Ultra-sonicated extraction conditions with regard to solvent (methanol, ethanol, 70% methanol) and time (30 min, 45 min, 60 min) were optimized according to the extraction efficiency of the total amount of the 12 analytes. For the extraction of experimental samples, the following conditions were chosen: 70% methanol was chosen as the solvent because it extracted a greater total of the 12 chemical components and because it gave better chromatographic resolution (Supplementary Materials Table S1). Two extractions were selected because two extractions yielded more than 95% of the total 12 chemical components, which was deemed acceptable. In the extractions of micro-dissected tissues, 70% methanol was also used; however, the extremely tiny size of the micro-dissected tissue samples and the tiny amount of extraction solvent meant that errors in operation could easily occur. Thus, the micro-dissected tissues were extracted only once, with 100 μL 70% methanol. This yielded an extraction ratio greater than 90%, which was also taken as acceptable.

### 2.2. Optimization of Analytical Conditions

**Figure 2:**
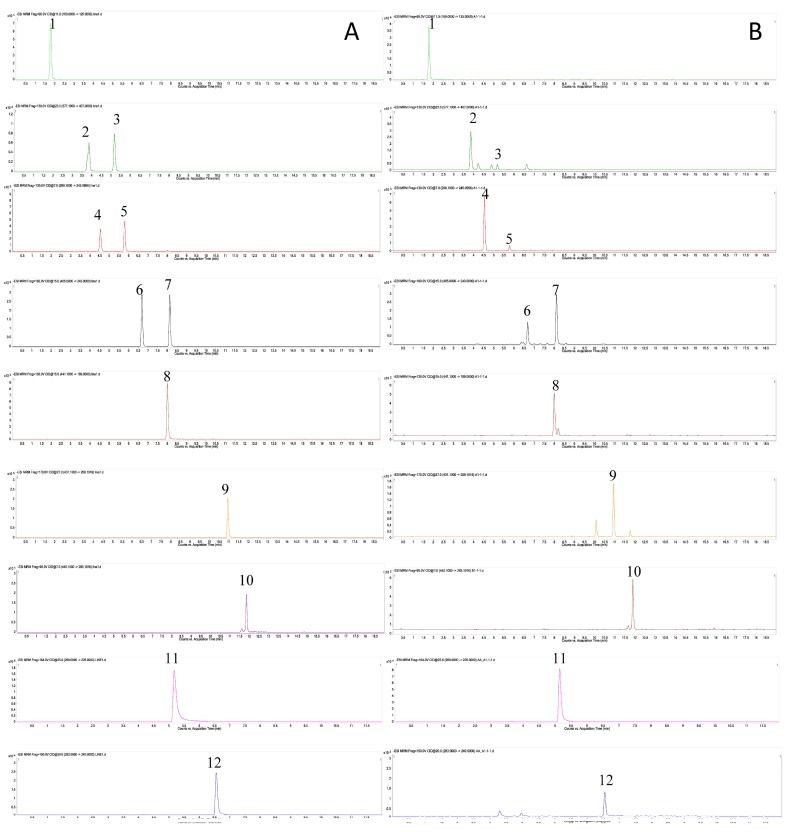
Mass spectrometry (MS) spectrums of 12 analytes with MRM mode. A: reference standard; B: sample (PMR-TA1). 1, gallic acid; 2, proanthocyanidin B1; 3, proanthocyanidin B2; 4, catechin; 5, epicatechin; 6, *cis*-THSG; 7, *trans*-THSG; 8, epicatechin-3-gallate; 9, emodin-8-*O*-β-D-glucoside; 10, physcion-8-*O*-β-D-glucoside; 11, Emodin; 12, Physcion.

In this study, UPLC-QqQ-MS/MS was used to explore the variation in content of the 12 major bioactive components between different grades and different tissues of PMR. In the optimization of UPLC-QqQ-MS/MS conditions, both negative and positive ion modes were tried, and negative ion mode was selected for its higher sensitivity (Supplementary Materials Table S1). The MRM fragments and collision voltage (eV) of each analyte were individually optimized by Mass Hunter Optimizer Software (Aglient Technologies, Inc., Santa Clara, CA, USA, 2010, Version B.03.01) (Table 1). For example, the ions [M − H]^−^ *m/z* 243.1 and *m/z* 173.1 of *trans*-THSG were observed under the MS condition of a 130 V fragmentor with the collision energy of 15 eV and 39 eV, respectively. Since the ion of *m/z* 243.1 was more in abundant, it was selected as the precursor ion of *trans*-THSG. The ion pairs of the other 11 analytes were similarly optimized. The MS spectrums of the analytes are shown in Figure 2. Two UPLC columns, ACQUITY HSS T3 (100 mm × 2.1 mm, 1.8 μm) and ACQUITY BEH (100 mm × 2.1 mm, 1.7 μm) [20], together with different mobile phase compositions (methanol-H_2_O/acetonitrile-H_2_O with or without acidic additive) were tested. For 10 chemical components of the 12 analytes, including gallic acid, proanthocyanidin B1, proanthocyanidin B2, *cis*-THSG, *trans*-THSG, catechin, epicatechin, epicatechin-3-gallate, emodin-8-*O*-β-D-glucoside, physcion-8-*O*-β-D-glucoside, better chromatographic resolution was achieved by the ACQUITY HSS T3 column using water containing 0.1% (*v*/*v*) formic acid and 0.1% (*v*/*v*) acetonitrile-formic acid as the mobile phases; whereas for emodin and physicon, better chromatographic resolution was obtained by the ACQUITY BEH column using 3 mM ammonium acetate in water and methanol. Based on the results, to provide suitable analytical conditions for all 12 analytes, two different conditions were used in this study.

### 2.3. Method Validation

As sample preparation procedures of raw material and tissue parts were different, the quantitative method was validated for both raw materials (PMR-RMA5) and micro-dissected tissues (PMR-TC2-5, PMR-TC3-6). As different tissues contain different chemical components, two tissue parts PMR-TC2-5 and PMR-TC3-6 were selected. For emodin-8-*O*-β-D-glucoside, physcion-8-*O*-β-D-glucoside, emodin, physcion, gallic acid and epicatechin-3-gallate, method validations were conducted on PMR-TC2-5; for the other six analytes, namely *cis*-THSG, *trans*-THSG, catechin, proanthocyanidin B1, proanthocyanidin B2, and epicatechin, method validations were conducted on PMR-TC3-6.

The methods of validation, including tests of linearity, limits of detection (LODs), limits of quantification (LOQs) are summarized in Table 1; precision, repeatability, stability and recovery of raw materials and micro-dissected tissues are summarized in Table 2 and Table 3, respectively. The results show good relationships between concentrations (R^2^ ≥ 0.9900). The LODs of all analytes are less than 17.95 ng/mL, while the LOQs are less than 48.25 ng/mL. The overall RSDs of intra-day and inter-day variations are not more than 7.48% and 9.84%, respectively. The spike recoveries of raw materials range from 82.51% to 107.37%, and the ranges of micro-dissected tissues are from 90.12% to 120.95%. Taking trace determination and the extremely small size of the samples into consideration, recovery results are acceptable. The need for stability is satisfied because the RSDs are no more than 9.00% in 48 h. All these results show that the established methods are accurate, sensitive, and precise for both raw materials and tissue-specific determination of all the 12 analytes in PMR.

### 2.4. Quantitative Results and Discussion

#### 2.4.1. Raw Materials

**Figure 3:**
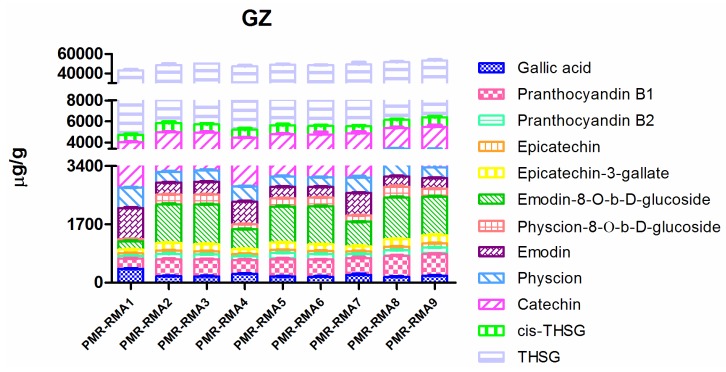
Content of the 12 analytes in raw material of Polygoni Multiflori Radix (*p* > 0.05).

Three major classes of chemicals, 12 compounds in total were quantitatively determined in raw materials of each grade of PMR samples, in order to explore the variation in major chemicals content of each grade and to find out whether there is a correlation between the existing grading system and the quality of the PMR. Sample extractions were first analyzed by UPLC-QqQ-MS/MS, and then one-way analysis of variance (ANOVA) statistical analysis was conducted by IBM SPSS Statistics 19.0 software (IBM, Armonk, NY, USA). The content of the 12 chemicals and variations are shown in Supplementary Materials Table S2 and Figure 3. In the statistical analysis, all *p*-values of the 12 analytes of each grade varied from 0.076 to 0.097. Since a *p*-value below 0.05 is taken as a significant difference between targeted groups, the results of statistical analysis in this study indicated that there were no significant differences among the three grades in the contents of the 12 chemical components. In detail, there were only slightly differences in the content of two stilbene glucosides, namely *trans*-THSG and *cis*-THSG. For example, the average contents of *trans*-THSG in the first grade, second grade and third grade were 42,018.66 ± 3115.35 μg/g, 42,853.91 ± 638.17 μg/g and 45,425.93 ± 1600.70 μg/g, respectively; and the *p*-value of three groups was 0.188 (>0.05). Similarly, the variations of the content of four anthraquinones, namely emodin, physcion, emodin-8-*O*-β-D-glucosides, physcion-8-*O*-β-D-glucosides, were only slight. For example, the average content of physcion was 414.95 ± 158.59 μg/g (grade 1), 343.84 ± 89.16 μg/g (grade 2) and 360.46 ± 80.12 μg/g (grade 3), and the *p*-value was 0.741 (>0.05). In the case of the six polyphenols, namely gallic acid, proanthocyanidin B1, proanthocyanidin B2, catechin, epicatechin, and epicatechin-3-gallate, the content of each grade also varied slightly. For example, the average content of catechin was 1559.21 ± 249.69 μg/g, 1670.31 ± 20.93 μg/g and 1995.95 ± 172.68 μg/g in the sequence of first grade, second grade and third grade; and *p*- was 0.076 (>0.05). Results revealed that PRM of different weights and sizes, which were recorded to be the basis for traditional quality grading [24,25], do not show any significant difference in chemical content. This fact suggests that the existing morphological criterion is unreliable for quality grading of PRM.

#### 2.4.2. Micro-Dissected Tissues

**Figure 4:**
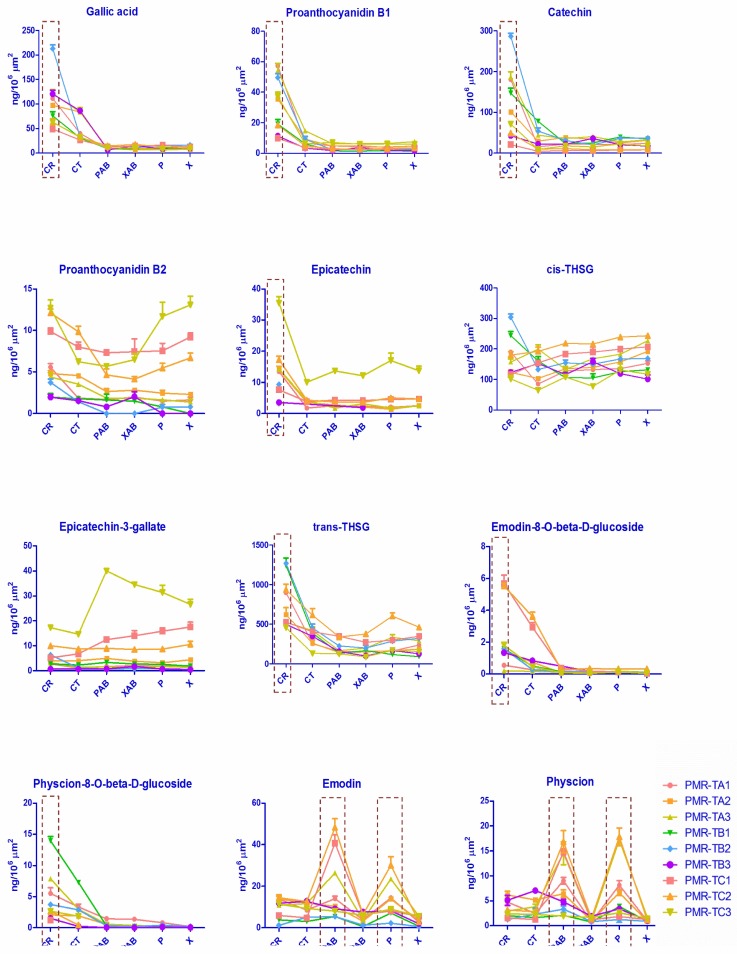
Variation in content of the 12 analytes in various tissues of PMR.

In order to further explore how quality could be linked with morphological features, tissue-specific chemical profiling was further conducted by a combined method of LMD and UPLC-QqQ-MS. Twelve major chemical components were quantitatively determined in all tissues of PMR. The varied amount of each chemical compound in all tissues is listed in Supplementary Materials Table S3 and graphed in Figure 4.

Results showed that the content of the 12 chemicals varied significantly in different tissues. For example, the content of *trans*-THSG of PMR-TA1 was 898.54 ± 15.48 ng/10^6^ μm^2^ in the cork; 255.06 ± 7.05 ng/10^6^ μm^2^ in the cortex; 154.51 ± 8.81 ng/10^6^ μm^2^ in the phloem of abnormal vascular bundles; 157.59 ± 15.05 ng/10^6^ μm^2^ in the xylem of abnormal vascular bundles; 163.41 ± 21.24 ng/10^6^ μm^2^ in the phloem; and 236.61 ± 13.04 ng/10^6^ μm^2^ in the xylem. In addition, the result of one-way ANOVA reveals that all *p*-values of the cork part were 0.00 (<0.05), which means the content of *trans*-THSG is significantly different to those of other tissues. The results indicate that *trans*-THSG mainly accumulated in the cork. This result is the same as our previous findings in histochemical study of PMR [26]. The same distribution pattern was found for the polyphenols: gallic acid (*p*-value 0.00), proanthocyanidin B1 (*p*-value 0.00), catechin (*p*-value 0.00), and epicatechin (*p*-value ≤ 0.019), and for the combined anthraquinones, namely emodin-8-*O*-β-D-glucosides (*p*-value ≤ 0.023) and physcion-8-*O*-β-D-glucosides (*p*-value 0.077 for cortex and *p*-value ≤ 0.01 for the other tissues). Two free anthraquinones: emodin and physcion presented a different tendency and were found to be mainly distributed in the phloem and abnormal phloem. For example, the content of emodin was 14.49 ± 1.39 ng/10^6^ μm^2^, 12.70 ± 0.89 ng/10^6^ μm^2^, 48.21 ± 4.26 ng/10^6^ μm^2^, 6.38 ± 0.82 ng/10^6^ μm^2^, 30.11 ± 4.18 ng/10^6^ μm^2^ and 2.97 ± 0.08 ng/10^6^ μm^2^ in the sequence of cork, cortex, phloem of abnormal vascular bundles, xylem of abnormal vascular bundles, phloem and xylem, respectively. Statistical analysis results have shown the *p*-value of physcion was no more than 0.038 and 0.006 in the phloem and abnormal phloem, respectively; and the *p*-value of emodin was no more than 0.022 in the abnormal phloem. This accumulated pattern differs from our previous findings, in which we proposed that the anthraquinones mainly exist in the cortex [26]. The inconsistency could be attributed to the limitations of tissue-separating technology at that time; tissues were separated manually and with adjoining tissues adhere, so tissues were easily mis-cut and, as a result, results were affected. In the case of the other three analytes: proanthocyanidin B2, epicatechin-3-gallate, and *cis*-THSG, the results of statistics analysis revealed that all *p*-values of these three analytes in different tissues were above 0.05, which indicates there were no obvious distribution patterns of these three chemical compounds. The results indicate that stilbene glucosides, combined anthraquinones and polyphenols were mainly accumulated in the cork; free anthraquinones mainly existed in the phloem, and thus the cork and phloem should be of more medicinal value than other tissues. The tissue-specific analysis of secondary metabolites creates a new morphological criterion for quality grading of PMR. That is, PMR with broader cork and phloem, as seen in a transverse section, were typically of better quality as these parts are where the bioactive components accumulate.

## 3. Materials and Methods

### 3.1. Plant Materials, Chemicals and Reagents

The PMR materials were collected from three different habitats in China, and three replicate sample materials were from different biological plants (Table 4). All samples were authenticated as the tuberous roots of *Polygonum multiflorum* Thunb. by Prof. Zhong-Zhen Zhao from the School of Chinese Medicine, Hong Kong Baptist University. Voucher specimens were deposited in the Bank of China (Hong Kong) Chinese Medicines Centre of Hong Kong Baptist University.

Chemical standards for *trans*-THSG, emodin, physcion, catechin, proanthocyanidin B1, proanthocyanidin B2, and gallic acid were purchased from Chengdu Must Bio-Technology Co., Ltd. (Chengdu, China); standards for *cis*-THSG, epicatechin, epicatechin-3-gallate, emodin-8-*O*-β-D-glucoside, and physcion-8-*O*-β-D-glucoside were purchased from Chengdu Xunchen Biological Technology Co., Ltd. (Chengdu, China). HPLC-grade methanol and acetonitrile (E. Merck, Darmstadt, Germany), ammonium acetate (Sigma-Aldrich, St. Louis, MO, USA), HPLC grade formic acid (Tedia, Fairfield, OH, USA) were purchased. Ultra-pure water was prepared by a Mili-Q water purification system (Millipore, Burlington, MA, USA).

### 3.2. Sample Preparation

#### 3.2.1. Raw Material Extraction

Raw materials were powdered, and each powdered sample of 0.1 g (accurately weighed) was ultra-sonicated with 20 mL 70% methanol for 45 min at 60 °C. Each solution was then centrifuged at 3000 rpm for 10 min. The supernatant was saved, and the residue was again extracted, and the supernatant saved. Then the residue was washed with 5 mL 70% methanol. The two supernatants and this solution were combined, made up to 50 mL with 70% methanol, and finally filtered through a 0.22 μm filter for quantitative analysis.

#### 3.2.2. Tissue Extraction

##### Laser Microdissection

PMR samples were first softened by wrapping with ultra-pure water-soaked non-cellulose paper, and then cut into small sections. Each section were then embedded in tissue-freezing medium (Thermo Shandon Limited, Runcorn, UK), and placed on a cutting platform in the cryostat (Thermo Shandon As620 Cryotome, UK) at −20 °C. Serial slices of 35 μm thickness were cut at −20 °C and directly moved on to a non-fluorescent positron-emission tomography (PET) microscope steel frame slide (76 mm × 26 mm, 4 μm thick, Leica, Wetzlar, Germany).

The slides were observed in fluorescence mode using a Leica microscope (Leica LMD 7000 system, Leica, Ben-shein, Germany). Laser microdissection was operated by a diode-pumped solid state (DPSS) laser beam at a speed of 1, aperture of 3, under a Leica LMD-BGR fluorescence filter system consisting of red light (excitation filter), blue light (suppression filter), green light (dichromatic mirror), with intensity of 50%, at 10× magnification. Six tissues, namely the cork, cortex, phloem of abnormal vascular bundles, xylem of abnormal vascular bundles, phloem and xylem were dissected under fluorescence inspection mode, within an area of approximately 1.5 × 10^6^ μm^2^. The micro-dissected tissues were collected into caps of 500 μL microcentrifuge tubes (Leica, Germany).

##### Micro-Dissected Tissue Extraction

The micro-dissected tissue in each cap was transferred to the bottom of a microcentrifuge tube by centrifugation (Centrifuge 5415R, Eppendorf, Hamburg, Germany) at 10,000 rpm for 5 min, and then was ultra-sonicated with 100 μL 70% methanol for 60 min, at 60 °C. Then the microcentrifuge tubes were centrifuged again for 10 min at 10,000 rpm. 90 μL of each supernatant was transferred to a glass insert with a plastic bottom spring (400 μL, Grace, Hong Kong, China) in a 1.5 mL brown HPLC vial (Grace, Hong Kong, China) and stored at 4 °C for quantitative analysis.

### 3.3. Ultra-Performance Liquid Chromatography Triple-Quadrupole Mass Spectrometry (UPLC-QqQ-MS/MS Analysis)

#### 3.3.1. Conditions

The quantitative analysis was conducted on an Agilent 6460 UHPLC-QqQ-MS/MS (Agilent Technologies, Santa Clara, CA, USA) with ESI ion source in negative mode. The chromatographic separations were acquired under two different conditions: (1) A UPLC T3 analytical column (2.1 mm × 100 mm, I.D. 1.8 μm, ACQUITY UPLC HSS, Waters, USA) coupled with a T3 pre-column (2.1 mm × 5 mm, I.D.1.8 μm, ACQUITY UPLC HSS, Waters, Milford, MA, USA) was used at 40 °C. The mobile phase consisted of (A) water and (B) acetonitrile, both containing 0.1% formic acid, and the gradient program was: 0 min, 5% B; 7 min, 20% B; 13 min, 50% B; 14.5 min, 100% B; 17 min, 100% B. The flow rate was 0.35 mL/min, and the injection volume was 2 μL. The source parameters were as follows: dry gas (N_2_) temperature 350 °C; flowrate 8 L/min; sheath gas flow 8 L/min with heater at 350 °C; nebulizer pressure, 45 psi; capillary voltage 3500 V, and dwell time of each ion pair 20 ms. (2) a UPLC C18 analytical column (2.1 mm × 100 mm, I.D. 1.7 μm, BEH) coupled with a C18 pre-column (2.1 mm × 5 mm, I.D.1.7 μm, ACQUITY UPLC BEH, Waters, USA) was used for chromatographic separations at 60 °C. The mobile phase consisted of 3 mM ammonium acetate in water (A) and methanol (B), and the gradient program was: 0 min, 35% B; 7 min, 100% B; 7–9 min, 100% B. The flow rate was 0.35 mL/min; the injection volume was 2 μL. The mass spectrometer parameters were set as: dry gas (N_2_) temperature 300 °C; flow rate 7 L/min; sheath gas flow 8 L/min; sheath gas heater 350 °C; nebulizer pressure, 45 psi; 500 V charging; capillary voltage 3500 V; and the dwell time for each ion pair was 40 ms. Other details are shown in Table 1. Each sample was analyzed in triplicate, and the final chemical contents of each analyte were calculated as an average ± standard deviation (SD) of those three readings.

#### 3.3.2. Quantitative Method Validation

Method validation in terms of linearity, LODs, LOQs, precision (intra-day and inter-day), repeatability, recovery and stability was carried out. The stock solutions of reference standards were diluted with methanol to yield a series of appropriate concentration solutions for assessing linearity, LODs (signal-to-noise (*S/N*) ratios of 3) and LOQs (*S/N* ratios of 10).

The precision validation of the assay method was calculated based on intra-day and inter-day variations. For intra-day variation assessment, six replicates of the PMR-RMA5 and micro-dissected tissues PMR-TC4-5 and PMR-TC7-6 were extracted and analyzed over the course of one day. For the inter-day variability test, the same samples were examined in triplicate on two consecutive days. Variations were expressed by the RSDs of the data (*n* = 6).

Method repeatability was evaluated by assessing variation in six replicated extracts of PMR-RMA5, PMR-TC2-5 and PMR-TC3-6. A stability test was performed on the sample solutions of PMR-RMA5, PMR-TC2-5 and PMR-TC3-6 over periods of 0, 2, 4, 8, 12, 24, 48 h after extraction, respectively.

As a recovery test for raw materials, 0.1 g of PMR-RMA5 was accurately weighed and different amounts (low, middle and high levels) of reference standards (gallic acid: 14.00, 18.00 and 22.00 μg; proanthocyanidin B1: 38.00, 48.00 and 57.00 μg; proanthocyanidin B2: 13.00, 16.00 and 19.00 μg; catechin: 130.00, 160.00 and 190.00 μg; epicatechin: 8.00, 10.00 and 12.00 μg; epicatechin-3-gallate: 18.00, 23.00 and 28.00 μg; *cis*-THSG:, and 32.00, 40.00, 48.00 μg; *trans*-THSG: 3.08, 3.85 and 4.62 mg; emodin-8-*O*-β-D-glucoside: 125.00, 156.00 and 187.00 μg; physcion-8-*O*-β-D-glucoside: 19.00, 24.00 and 29.00 μg; emodin: 30.00, 38.00 and 46.00 μg; physcion: 25.00, 32.00 and 38.00 μg, repectively) were spiked, and then extracted and analyzed in triplicate.

To assess the recovery for micro-dissected tissues, tissues with known contents of the 12 analytes were cut by LMD with an area of 1.5 × 10^6^ μm^2^, and different amounts (low, middle and high levels) of reference standards (gallic acid: 90, 110 and 130 ng; proanthocyanidin B1: 40, 50 and 60 ng; proanthocyanidin B2: 110, 140 and 170 ng; catechin: 200, 250 and 300 ng; epicatechin: 100, 125 and 150 ng; epicatechin-3-gallate: 120, 150 and 180 ng; *cis*-THSG: 2300, 2800 and 3300 ng; *trans*-THSG: 3600, 4400 and 5200 ng; emodin-8-*O*-β-D-glucoside: 150, 190 and 230 ng; physcion-8-*O*-β-D-glucoside: 22, 27 and 32 ng; emodin: 150, 180 and 210 ng; physcion: 80, 100 and 120 ng, in solution, respectively) were spiked, then extracted and analyzed in triplicate for each level.

### 3.4. Data Analysis

The quantitative data obtained were processed by Agilent Mass Hunter Work station software-Quantitative Analysis (version B.06.00, Build 6.0.388.1, Agilent Technologies Inc., Santa Clara, CA, USA, 2008). Data were presented as mean ± SD of triplicate determinations. To compare the significant differences between the content of active compounds in the samples, one-way ANOVA was conducted by IBM SPSS Statistics 19.0 software (IBM, USA). A significant difference is indicated by *p* values.

## 4. Conclusions

In this study, a combination of LMD and UHPLC-QqQ-MS/MS was applied for histochemical analysis of different commercial grades of PMR, both raw material and individual tissues, in order to find the scientific basis for grading classification. UHPLC-QqQ-MS/MS was used to determine quantitatively the content of 12 major bioactive components of PMR raw materials firstly, and then LMD coupled with UHPLC-QqQ-MS/MS was used to determine and find the distribution patterns of three major types of chemicals (12 analytes) in different tissues of PMR. Results indicated that traditional commercial grades based on size and weight of PRM did not correspond to any significant difference in chemical content. Interestingly, the tissue-specific analysis revealed a strong and consistent relationship between phytochemicals and histologic structures. In particular, stilbene glucosides, combined anthraquinones and polyphenols, were mainly distributed in the cork, and free anthraquinones were mainly distributed in the phloem. Thus, PMR with broader cork and phloem, as seen in a transverse section, were typically of better quality as these parts are where the bioactive components accumulated. The tissue-specific analysis of secondary metabolites creates a reliable morphological criterion for the quality grading of PMR.

## Figures and Tables

**Table 1:** Multiple reaction monitoring (MRM) conditions in ultra-performance liquid chromatography triple-quadrupole mass spectrometry (UPLC-QqQ-MS/MS) analysis and method validation (linearity, limits of detection (LODs), limits of quantification (LOQs)) for quantitative determination of secondary metabolites.

Analyte	MRM	Collision Voltage (eV)	Calibration Curve			Sensitivity (ng/mL)	
			Range (ng/mL)	Equation	R^2^	LODs	LOQs
Gallic acid	169.0→125.0	11	80–4000	y = 95.80x − 605.88	0.9972	4.12	14.85
Proanthocyanidin B1	577.1→407.0	23	40–2000	y = 28.09x − 105.54	0.9994	2.51	7.59
Catechin	289.1→245.1	7	20–1000	y = 25.33x + 256.56	0.9974	17.95	48.25
Proanthocyanidin B2	577.1→407.0	23	40–2000	y = 25.93x − 48.90	0.9991	3.17	15.92
Epicatechin	289.1→245.1	7	10–1000	y = 31.33x − 78.60	0.9939	15.52	40.66
*cis*-THSG	405.0→243.0	15	5–1000	y = 213.54x − 348.14	0.9948	1.04	1.62
Epcatechini-3-gallate	441.1→169.0	15	5–1000	y = 55.85x − 82.32	0.9936	0.99	6.62
*trans*-THSG	405.0→243.1	15	40–8000	y = 196.23x − 357.59	0.9992	9.64	11.02
Emodin-8-*O*-β-D-glucoside	431.1→269.1	27	40–2000	y = 801.51x + 29.72	0.9911	4.36	4.58
Physcion-8-*O*-β-D-glucoside	445.1→283.1	7	40–2000	y = 58.92x − 103.32	0.9929	4.84	7.14
Emodin	269.0→225.0	25	80–800	y = 2422.72 + 16798.78	0.9907	0.22	1.15
Physcion	283.0→240.0	20	40–2000	y = 117.20x − 1082.86	0.9945	1.73	4.03

**Table 2:** Method validation (repeatability, precision, spike recovery and stability) for quantitative determination of secondary metabolites in raw materials of PMR.

	Repeatability (*n* = 6, RSD, %)	Precision (*n* = 6)				Spike Recovery (*n* = 3, Mean (RSD), %)			Stability (48 h, *n* = 7)	
		Intra-day		Inter-Day		Low	Middle	High		
		Mean (ng/mL)	RSD, %	Mean (ng/mL)	RSD, %				Mean (ng/mL)	RSD, %
Gallic acid	10.81	394.15	3.01	471.86	7.69	96.71 (4.58)	97.23 (4.15)	94.94 (1.53)	445.58	8.73
Proanthocyanidin B1	7.43	959.60	2.47	999.00	4.08	90.73 (6.29)	91.72 (5.46)	90.85 (4.85)	1042.45	7.81
Catechin	9.35	293.40	4.43	329.87	9.01	101.89(7.75)	95.14(6.51)	98.52(1.86)	315.646	7.38
Proanthocyanidin B2	8.31	341.51	2.21	357.12	4.16	96.17(4.73)	93.72(1.52)	96.08(3.70)	369.96	5.54
Epicatechin	3.48	205.35	5.58	221.47	4.09	102.69(4.13)	107.37(1.31)	101.70(4.44)	203.26	5.68
*cis*-THSG	4.22	2012.47	1.21	2198.07	1.79	82.51(10.18)	102.87(9.21)	87.96(10.49)	2107.79	4.76
Epcatechini-3-gallate	7.27	440.67	7.25	448.75	5.69	103.50(5.13)	95.52(8.18)	100.89(2.06)	456.16	4.23
*trans*-THSG	2.42	7396.96	2.28	7932.62	6.72	95.15(6.80)	92.82(5.34)	85.98(8.56)	7865.36	5.95
Emodin-8-*O*-β-D-glucoside	3.19	2124.69	0.48	2365.74	0.78	94.72(1.47)	100.49(1.99)	97.93(1.58)	2272.80	4.48
Physcion-8-*O*-β-D-glucoside	3.58	503.18	1.54	558.48	1.59	94.11(2.09)	95.36(4.40)	85.81(0.38)	529.89	4.66
Emodin	2.02	719.23	8.32	669.05	9.95	99.05(2.92)	106.78(2.56)	101.18(2.33)	682.54	5.77
Physcion	3.40	605.38	4.49	569.70	6.19	97.94(9.05)	92.87(6.61)	99.27(8.78)	578.33	3.97

**Table 3:** Method validation (repeatability, precision, spike recovery and stability) for quantitative determination of secondary metabolites in micro-dissected tissues of PMR.

	Repeatability (*n* = 6, RSD, %)	Precision (*n* = 6)				Spike Recovery (*n* = 3, mean (RSD), %)			Stability (48 h, *n* = 7)	
		Intra-Day		Inter-day		Low	Middle	High		
		Mean (ng/mL)	RSD, %	Mean (ng/mL)	RSD, %				Mean (ng/mL)	RSD, %
Gallic acid	2.85	144.60	3.08	147.10	8.14	108.78(5.00)	101.82(15.80)	120.95(10.18)	142.90	3.82
Proanthocyanidin B1	9.55	54.92	5.65	60.05	4.27	111.34(11.31)	109.33(10.52)	111.09(4.81)	51.82	5.19
Catechin	6.11	113.16	7.96	141.29	6.68	109.47(5.94)	102.63(11.29)	98.44(5.67)	135.61	5.60
Proanthocyanidin B2	6.25	96.75	6.14	103.44	6.50	100.39(11.61)	99.32(3.13)	100.73(0.33)	100.70	7.46
Epicatechin	3.68	63.25	7.48	72.78	4.76	100.98(7.78)	100.80(9.17)	109.27(9.68)	78.55	8.64
cis-THSG	4.25	3040.38	2.09	3682.49	3.91	113.93(9.18)	103.54(6.08)	99.46(5.65)	3378.57	5.82
Epcatechini-3-gallate	6.33	162.27	6.03	178.00	4.79	93.66(8.48)	97.62(6.72)	90.98(10.54)	131.54	4.90
trans-THSG	5.91	4296.71	0.56	4921.06	5.25	109.77(6.74)	112.86(4.00)	102.28(9.76)	7249.63	7.37
Emodin-8-*O*-β-D-glucoside	4.92	193.50	2.44	241.92	3.34	113.83(1.50)	109.74(15.73)	101.83(2.13)	359.83	6.05
Physcion-8-*O*-β-D-glucoside	11.17	25.00	6.44	31.79	9.84	109.61(6.19)	103.38(8.95)	115.32(8.42)	28.27	9.00
Emodin	6.23	183.42	1.66	192.12	4.33	102.25(8.27)	107.03(11.56)	109.24(1.84)	218.22	8.14
Physcion	11.22	90.94	1.92	85.36	5.69	94.96(4.87)	90.12(9.48)	92.84(6.01)	68.27	3.54

**Table 4:** Sample information of PMR.

Sample No.	Grade	Type ^(a)^	Cultivated Location	Collection Date
PMR-RMA1	1 ^(b)^	RM	Guizhou Province, Shibing Country	2017-1-18
PMR-RMA2	1	RM	Guizhou Province, Shibing Country	2017-1-16
PMR-RMA3	1	RM	Guizhou Province, Shibing Country	2017-2-18
PMR-RMA4	2	RM	Guizhou Province, Shibing Country	2017-1-18
PMR-RMA5	2	RM	Guizhou Province, Shibing Country	2017-2-16
PMR-RMA6	2	RM	Guizhou Province, Shibing Country	2017-2-18
PMR-RMA7	3	RM	Guizhou Province, Shibing Country	2017-1-18
PMR-RMA8	3	RM	Guizhou Province, Shibing Country	2017-2-16
PMR-RMA9	3	RM	Guizhou Province, Shibing Country	2017-2-18
PMR-TA1	1	T	Guizhou Province, Shibing Country	2017-2-16
PMR-TA2	2	T	Guizhou Province, Shibing Country	2017-2-16
PMR-TA3	3	T	Guizhou Province, Shibing Country	2017-2-16
PMR-TB1	1	T	Guangdong Province, Xinxing Country	2017-2-8
PMR-TB2	2	T	Guangdong Province, Xinxing Country	2017-2-8
PMR-TB3	3	T	Guangdong Province, Xinxing Country	2017-2-8
PMR-TC1	1	T	Hubei Province, Lizhou City	2017-1-16
PMR-TC2	2	T	Hubei Province, Lizhou City	2017-1-16
PMR-TC3	3	T	Hubei Province, Lizhou City	2017-1-16

(a) RM: sample used for raw material analysis; T: used for micro-dissected tissue analysis; all samples were 1 year cultivated ones; (b) Grade 1: >100 g; grade 2: 50–100 g; grade 3: <50 g.

## Supplementary Materials

Table S1: Method optimization for quantification of secondary metabolites Table S2: Quantitative results of secondary metabolites in raw materials of PMR (μg·g^−1^) (*n* = 3). Table S3: Quantitative results of secondary metabolites in micro-dissected tissues of PMR (ng/106 μm^2^) (*n* = 3).
