# Supplementary material for: Tissue-Specific Analysis of Secondary Metabolites Creates a Reliable Morphological Criterion for Quality Grading of Polygoni Multiflori Radix
Source: Molecules. 2018 May 8;23(5):1115. doi: 10.3390/molecules23051115 (PMC6099783; doi:10.3390/molecules23051115)
Supplement: Supplementary file 1 [file molecules-23-01115-s001.pdf]

**Table S1 Method optimization for quantification of secondary metabolites**

| **Secondary metabolites** | **Ion mode** | | | | **Extraction solvents** | | |
|---|---|---|---|---|---|---|---|
| | Positive | | Negative | | Ethanol (peak area) | Methanol (peak area) | 70% methanol (peak area) |
| | Ion pairs | abundance | Ion pairs | abundance | | | |
| Gallic acid | -a | -a | 169.0→125.0 | 43290 | 1081 | 14414 | 16916 |
| Proanthocyanidin B1 | 579.2→73.0 | 40589 | 577.1→407.0 | 16084 | 26621 | 77828 | 74614 |
| Catechin | 291.1→139..1 | 33504 | 289.1→245.1 | 134961 | 130703 | 190111 | 179497 |
| Proanthocyanidin B2 | 579.2→73.1 | 32829 | 577.1→407.0 | 53145 | 10451 | 13187 | 12183 |
| Epicatechin | 291.1→139.1 | 336451 | 289.1→245.1 | 46459 | 5436 | 8304 | 7496 |
| *cis*-THSG | -b | -b | -b | -b | 100538 | 106339 | 112458 |
| Epcatechini-3-gallate | 443.1→123.0 | 108037 | 441.1→169.0 | 158778 | 803693 | 205802 | 789209 |
| *trans*-THSG | 407.1→245.1 | 971191 | 405.0→243.0 | 778569 | 15209041 | 16725440 | 17105686 |
| Emodin-8-*O*-β-D-glucoside | 433.1→365.1 | 856 | 431.1→269.1 | 835457 | 2810316 | 2576490 | 3197349 |
| Physcion-8-*O*-β-D-glucoside | 447.1→207.1.1 | 13145 | 445.1→283.1 | 26439 | 54205 | 36351 | 62959 |
| Emodin | 271.1→115.0 | 15371 | 269.0→225.0 | 255952 | 366282 | 504696 | 559892 |
| Physcion | 285.1→139.0 | 35857 | 283.0→240.0 | 17876 | 30143 | 36044 | 61706 |
| total | - | 1587830 | - | 2367010 | 19548507 | 20495004 | 22179962 |

Data in the table is an average of duplicate. a) No ion pair was found under the positive mode; b) for the ion mode selection, *cis*-THSG was referenced with its isomer *trans*-THSG.

**Table S2 Quantitative results of secondary metabolites in raw materials of PMR (μg·g$^{-1}$) (n=3)**

| Secondary metabolites | Mean (RSD %) | | | | | | | | | One-way ANOVA *P* value |
|---|---|---|---|---|---|---|---|---|---|---|
| | PMR-RMA1 | PMR-RMA2 | PMR-RMA3 | PMR-RMA4 | PMR-RMA5 | PMR-RMA6 | PMR-RMA7 | PMR-RMA8 | PMR-RMA9 | |
| Gallic acid | 402.07(1.80) | 186.06(4.06) | 182.35(6.37) | 253.46(4.44) | 179.23(4.98) | 166.29(9.04) | 227.60(11.04) | 162.81(7.98) | 197.92(2.10) | 0.605 |
| Proanthocyanidin B1 | 295.02(4.25) | 490.67(3.16) | 493.72(2.55) | 406.08(3.11) | 502.83(4.49) | 507.67(0.43) | 485.89(4.23) | 611.76(3.15) | 635.29(0.80) | 0.173 |
| Catechin | 1274.44(1.83) | 1740.64(2.05) | 1662.55(4.98) | 1647.71(0.94) | 1689.03(3.20) | 1674.20(13.81) | 1781.35(9.49) | 1959.84(4.14) | 2126.65(7.37) | 0.076 |
| Proanthocyanidin B2 | 84.26(7.14) | 160.86(5.68) | 150.71(1.71) | 111.53(6.27) | 179.71(6.11) | 159.64(0.75) | 115.88(9.61) | 168.66(5.34) | 193.97(2.81) | 0.693 |
| Epicatechin | 71.56(13.22) | 98.09(7.06) | 88.80(5.61) | 65.12(5.34) | 89.49(3.05) | 88.93(2.46) | 76.48(15.14) | 105.27(5.21) | 113.88(8.29) | 0.438 |
| *cis*-THSG | 684.52(8.66) | 870.18(13.59) | 772.65(10.00) | 759.46(16.08) | 847.04(11.74) | 828.27(8.72) | 685.83(10.79) | 781.87(9.23) | 901.23(10.12) | 0.880 |
| Epcatechini-3-gallate | 108.96(5.21) | 225.90(1.33) | 205.48(3.92) | 144.92(3.58) | 224.15(1.91) | 195.86(1.31) | 154.64(6.00) | 225.56(3.28) | 246.99(2.78) | 0.783 |
| *trans*-THSG | 38539.11(2.30) | 42967.88(2.63) | 44548.99(0.13) | 42211.42(2.21) | 43487.67(1.24) | 42862.64(0.51) | 43803.45(4.80) | 45470.41(1.22) | 47003.93(2.17) | 0.188 |
| Emodin-8-*O*-β-D-glucoside | 248.69(5.21) | 1120.62(1.76) | 1159.35(0.36) | 582.48(1.06) | 1042.90(1.70) | 1104.73(1.39) | 717.48(0.66) | 1206.13(1.26) | 1122.51(0.69) | 0.852 |
| Physcion-8-*O*-β-D-glucoside | 60.26(6.46) | 288.31(2.42) | 288.16(4.69) | 141.12(2.46) | 240.22(3.62) | 255.17(2.39) | 180.54(4.60) | 314.43(8.07) | 229.08(0.94) | 0.907 |
| Emodin | 902.99(2.19) | 349.57(1.46) | 371.48(1.85) | 652.66(2.49) | 332.25(2.36 | 312.93(1.54) | 653.89(2.05) | 302.52(4.31) | 309.05(2.65) | 0.805 |
| Physcion | 597.68(1.39) | 313.10(4.98) | 334.08(5.86) | 445.46(1.33) | 307.35(4.92) | 278.71(4.23) | 452.86(6.71) | 318.08(11.01) | 310.43(13.22) | 0.741 |

Data in the table is an average of triplicate, and the numbers in parentheses/brackets are the RSD values in % of triplicate.

**Table S3 Quantitative results of secondary metabolites in micro-dissected tissues of PMR (ng/$10^6$ μ$m^2$) (n=3)**

| Secondary metabolites | micro-dissected tissues | Mean (RSD %) | | | | | | | | |
|---|---|---|---|---|---|---|---|---|---|---|
| | | PMR-TA1 | PMR-TA2 | PMR-TA3 | PMR-TB1 | PMR-TB2 | PMR-TB3 | PMR-TC1 | PMR-TC2 | PMR-TC3 |
| **gallic acid** | CR | 110.97(3.66) | 97.42(4.09) | 121.34(6.40) | 75.63(11.43) | 213.00(3.65) | 120.30(6.63) | 48.82(6.87) | 63.04(10.95) | 62.98(1.98) |
| | CT | 39.94(4.26) | 83.67(1.20) | 83.27(11.17) | 30.67(14.86) | 33.25(13.72) | 86.27(1.51) | 26.29(5.08) | 31.69(2.84) | 32.46(3.64) |
| | XAB | 7.73(9.35) | 9.28(10.57) | 7.69(14.37) | 9.16(4.16) | 9.16(18.05) | 6.51(22.77) | 12.57(3.44) | 14.69(2.31) | 9.86(2.07) |
| | PAB | 8.01(18.61) | 6.39(6.11) | 9.39(14.20) | 9.11(8.47) | 10.88(11.11) | 15.40(13.54) | 14.47(6.45) | 17.48(2.63) | 10.23(2.62) |
| | X | 10.13(3.25) | 6.88(1.20) | 6.14(8.08) | 10.68(7.11) | 15.65(7.70) | 8.18(27.13) | 15.95(16.12) | 12.68(2.37) | 7.37(11.28) |
| | P | 13.95(3.43) | 7.84(6.43) | 7.82(10.52) | 8.58(1.87) | 16.44(2.66) | 9.51(12.34) | 12.37(2.93) | 12.49(2.16) | 8.82(21.04) |
| **proanthocyanidin B1** | CR | 57.23(0.92) | 35.49(2.20) | 54.25(8.59) | 19.11(16.34) | 49.64(5.42) | 11.25(1.94) | 9.86(3.06) | 18.43(2.44) | 37.27(7.45) |
| | CT | 6.66(6.28) | 9.14(2.95) | 14.74(9.87) | 6.21(9.77) | 9.14(20.65) | 3.34(10.99) | 3.62(10.94) | 4.92(10.99) | 6.27(2.12) |
| | XAB | 4.82(4.16) | 4.78(6.07) | 6.26(6.35) | 1.52(18.30) | 2.28(6.38) | 1.74(34.18) | 2.41(3.15) | 3.06(17.79) | 7.02(0.61) |
| | PAB | 4.27(24.19) | 4.89(2.86) | 6.35(5.69) | 1.34(17.23) | 2.15(9.37) | 3.66(7.88) | 2.39(9.35) | 2.39(14.75) | 5.98(5.59) |
| | X | 4.18(17.36) | 3.86(5.70) | 6.46(9.45) | 1.76(12.11) | 2.66(15.73) | 1.89(11.41) | 2.38(19.70) | 3.23(3.27) | 6.11(16.74) |
| | P | 4.66(5.33) | 4.83(8.28) | 7.58(8.13) | 1.28(18.82) | 2.59(6.54) | 1.75(17.69) | 3.41(5.48) | 3.48(1.15) | 5.90(6.04) |
| **catechin** | CR | 180.76(2.13) | 101.37(2.87) | 186.85(7.00) | 147.70(8.00) | 287.19(2.66) | 43.10(7.18) | 21.23(5.45) | 48.75(4.96) | 71.46(3.73) |
| | CT | 15.67(9.78) | 29.40(1.18) | 44.19(6.65) | 78.38(3.87) | 54.06(15.52) | 22.46(1.99) | 4.62(4.25) | 10.27(9.46) | 9.24(8.09) |
| | XAB | 22.36(10.71) | 38.57(4.02) | 35.98(3.01) | 23.29(8.05) | 31.01(7.92) | 22.26(9.54) | 6.11(3.27) | 11.11(8.54) | 17.90(2.26) |
| | PAB | 21.60(17.15) | 34.44(4.30) | 40.69(7.84) | 25.31(2.73) | 20.60(86.86) | 35.83(6.87) | 6.39(5.01) | 8.59(8.50) | 15.13(3.57) |
| | X | 20.97(6.61) | 25.16(7.30) | 27.60(8.05) | 39.92(5.84) | 36.35(6.76) | 20.64(8.95) | 7.66(2.50) | 8.67(5.15) | 22.62(10.25) |
| | P | 24.26(3.80) | 31.81(5.30) | 32.55(3.85) | 34.56(6.07) | 36.79(4.07) | 17.67(9.32) | 7.98(7.18) | 8.56(6.90) | 18.19(7.18) |
| **proanthocyanidin B2** | CR | 5.57(7.90) | 4.82(7.20) | 4.40(12.10) | 1.97(22.50) | 3.75(10.65) | 1.96(16.05) | 9.87(4.24) | 12.11(4.01) | 12.68(7.86) |

| | | | | | | | | | | |
|---|---|---|---|---|---|---|---|---|---|---|
| | CT | 1.87(13.37) | 4.57(4.51) | 3.53(4.67) | 1.78(7.66) | 1.43(8.90) | 1.55(9.93) | 8.02(7.53) | 9.85(6.45) | 6.24(3.29) |
| | XAB | 1.70(15.29) | 2.65(14.18) | 1.87(11.19) | * | * | 0.81(19.84) | 7.31(4.88) | 4.66(15.15) | 5.70(4.65) |
| | PAB | 1.98(10.10) | 2.81(7.02) | 1.85(5.05) | 1.49(12.04) | * | * | 7.44(20.66) | 4.15(7.73) | 6.50(10.63) |
| | X | 1.51(16.56) | 2.47(13.27) | 1.66(10.38) | * | * | * | 7.54(11.86) | 5.48(10.39) | 11.69(14.65) |
| | P | 1.63(21.47) | 2.32(5.07) | 1.38(24.65) | * | 0.81(10.43) | * | 9.21(5.29) | 6.71(8.45) | 13.09(7.83) |
| **epicatechin** | CR | 13.49(1.88) | 14.22(6.55) | 14.23(4.86) | 3.10(17.42) | 9.37(7.36) | 3.54(7.86) | 7.65(5.10) | 17.30(6.59) | 35.48(5.74) |
| | CT | 1.75(19.05) | 3.65(8.46) | 4.24(8.42) | * | * | * | 3.83(15.57) | 4.32(3.49) | 10.05(8.04) |
| | XAB | 2.60(9.18) | 2.74(17.79) | * | * | * | * | 4.29(6.79) | 3.75(5.36) | 13.73(2.55) |
| | PAB | 2.35(4.48) | 2.12(11.01) | 3.16(9.52) | * | * | 1.90(5.95) | 4.18(8.46) | 3.65(5.45) | 12.15(5.11) |
| | X | 2.01(2.73) | * | 1.77(3.32) | * | * | * | 4.59(8.87) | 5.01(0.81) | 17.05(13.84) |
| | P | 2.49(15.33) | 2.54(23.02) | 2.45(18.77) | * | * | * | 4.69(5.71) | 4.70(8.84) | 13.76(10.53) |
| ***cis*-THSG** | CR | 189.76(4.08) | 123.52(6.57) | 157.43(6.20) | 244.08(5.52) | 304.24(3.33) | 123.34(3.20) | 116.48(6.11) | 180.27(6.01) | 102.23(11.45) |
| | CT | 85.39(4.34) | 103.89(2.67) | 199.38(7.13) | 160.94(8.80) | 133.03(10.09) | 152.86(1.35) | 154.82(2.19) | 192.25(7.27) | 65.39(1.60) |
| | XAB | 128.35(6.52) | 135.58(0.65) | 133.87(6.70) | 105.94(16.36) | 155.29(5.71) | 116.16(3.90) | 183.62(3.74) | 218.33(1.52) | 108.47(2.17) |
| | PAB | 132.48(11.93) | 140.21(5.58) | 168.02(4.38) | 105.53(12.99) | 150.38(13.10) | 158.59(6.77) | 190.32(8.24) | 216.64(2.55) | 78.09(0.86) |
| | X | 136.10(5.57) | 157.74(3.50) | 183.04(4.59) | 125.80(6.58) | 167.50(3.53) | 119.39(8.87) | 199.40(2.51) | 239.20(1.19) | 131.07(15.63) |
| | P | 153.33(5.04) | 192.25(2.53) | 227.34(1.85) | 131.90(1.83) | 168.83(2.04) | 101.65(13.51) | 207.53(1.92) | 243.56(1.09) | 117.72(2.02) |
| **epicatechin-3-gallate** | CR | 2.81(7.74) | 3.99(4.31) | 3.18(14.24) | 2.83(12.28) | 6.36(3.31) | 0.66(14.24) | 5.16(2.99) | 10.01(6.74) | 17.26(1.90) |
| | CT | 1.12(10.63) | 4.00(2.11) | 1.38(20.97) | 2.26(10.92) | 1.07(10.66) | 0.69(24.35) | 6.76(2.51) | 8.69(10.74) | 14.68(4.44) |
| | XAB | 1.69(9.66) | 4.93(14.68) | 1.56(10.58) | 3.30(16.39) | 0.70(18.50) | 0.75(13.36) | 12.47(6.99) | 8.94(7.87) | 40.08(1.71) |
| | PAB | 2.06(24.08) | 3.87(7.75) | 1.36(6.90) | 2.70(25.19) | 0.84(31.40) | 1.74(5.14) | 14.01(14.96) | 8.55(10.13) | 34.56(2.58) |
| | X | 1.78(15.29) | 3.20(1.17) | 1.72(13.88) | 2.56(3.51) | 0.87(4.60) | 0.71(16.40) | 15.88(8.07) | 8.68(4.22) | 31.38(9.42) |
| | P | 1.97(5.23) | 4.39(8.03) | 1.35(19.26) | 1.89(14.24) | 0.57(18.43) | 0.50(10.38) | 17.56(11.11) | 10.59(11.52) | 26.65(7.45) |

| | | | | | | | | | | |
|---|---|---|---|---|---|---|---|---|---|---|
| ***trans*-THSG** | CR | 898.54(1.72) | 621.95(13.99) | 522.94(3.82) | 1242.26(7.65) | 1271.63(1.58) | 499.75(3.10) | 528.15(5.20) | 936.45(7.57) | 457.52(4.41) |
| | CT | 255.06(2.76) | 262.00(15.07) | 421.98(10.50) | 387.70(8.68) | 453.24(10.77) | 349.04(0.27) | 406.06(4.85) | 616.05(13.46) | 133.58(1.11) |
| | XAB | 154.51(5.57) | 143.40(4.25) | 165.36(7.57) | 132.83(9.35) | 225.86(8.57) | 155.85(12.88) | 348.95(6.08) | 339.33(2.10) | 126.00(0.79) |
| | PAB | 157.59(9.55) | 152.16(7.60) | 200.27(12.70) | 165.02(2.68) | 199.77(16.45) | 98.94(8.57) | 273.09(8.57) | 378.08(3.71) | 87.88(2.98) |
| | X | 163.41(13.00) | 167.40(5.00) | 336.13(9.46) | 117.43(7.66) | 285.47(9.88) | 167.65(11.12) | 300.80(6.64) | 603.67(6.46) | 171.76(1.40) |
| | P | 236.61(5.51) | 187.20(7.74) | 291.68(6.64) | 90.80(13.65) | 322.83(1.40) | 125.64(10.13) | 348.36(3.31) | 462.93(0.19) | 153.76(3.31) |
| **emodin-8-*O*-β-D-glucoside** | CR | 0.55(7.27) | 1.47(8.00) | 0.20(7.15) | 1.58(4.03) | 1.59(7.88) | 1.34(3.18) | 5.68(9.33) | 5.54(3.10) | 1.82(3.15) |
| | CT | 0.27(7.41) | 0.73(6.56) | 0.17(11.02) | 0.50(20.63) | 0.28(12.37) | 0.83(1.99) | 2.93(9.80) | 3.62(7.33) | 0.46(3.24) |
| | XAB | 0.19(10.53) | 0.08(12.20) | 0.11(6.40) | 0.15(17.08) | 0.14(3.70) | * | 0.36(19.34) | 0.25(12.60) | 0.09(6.26) |
| | PAB | 0.09(11.11) | 0.05(18.15) | 0.08(16.97) | 0.11(26.61) | 0.14(20.33) | 0.16(13.09) | 0.18(9.53) | 0.34(3.07) | 0.06(13.96) |
| | X | 0.14(21.43) | 0.13(4.48) | 0.18(10.47) | 0.12(11.15) | 0.09(12.96) | * | 0.14(11.49) | 0.31(2.02) | 0.12(8.84) |
| | P | 0.05(20.00) | 0.05(23.50) | 0.12(11.27) | 0.06(14.01) | 0.09(2.06) | * | 0.04(9.51) | 0.32(12.67) | 0.05(16.29) |
| **physcion-8-*O*-β-D-glucoside** | CR | 5.52(16.54) | 2.07(11.07) | 7.86(2.59) | 13.97(4.87) | 3.75(2.22) | 1.54(5.07) | 1.26(23.62) | 2.67(10.81) | 2.67(6.74) |
| | CT | 3.36(13.13) | 1.92(7.45) | 3.01(2.63) | 7.30(4.86) | 2.90(2.13) | 0.24(25.57) | * | 0.45(10.10) | 1.86(6.05) |
| | XAB | 1.46(9.15) | 0.59(20.54) | 0.58(14.47) | 0.30(8.45) | 0.36(24.66) | 0.06(23.86) | * | * | * |
| | PAB | 1.37(14.24) | 0.08(33.03) | 0.38(23.23) | 0.22(15.74) | 0.23(9.86) | * | * | * | * |
| | X | 0.82(15.15) | 0.28(13.96) | 0.28(12.33) | 0.17(22.86) | 0.45(4.44) | 0.11(24.70) | * | * | * |
| | P | 0.22(13.03) | * | 0.15(8.60) | * | 0.23(31.40) | * | * | * | * |
| **emodin** | CR | 13.84(9.39) | 13.86(8.92) | 10.99(6.23) | 3.81(9.68) | 1.21(8.80) | 11.86(12.13) | 5.95(7.15) | 14.49(.63) | 11.47(8.16) |
| | CT | 8.57(4.57) | 12.00(1.29) | 11.71(5.33) | 3.13(21.58) | 5.08(8.73) | 12.82(2.17) | 4.85(11.62) | 12.70(7.04) | 9.32(2.50) |
| | XAB | 14.07(8.46) | 10.38(3.49) | 26.45(2.03) | 5.45(12.50) | 5.45(15.98) | 9.15(5.00) | 40.75(9.61) | 48.21(8.84) | 8.05(1.96) |
| | PAB | 3.95(18.15) | 3.25(8.54) | 3.89(13.00) | 0.68(24.60) | 1.33(32.58) | 7.68(6.26) | 6.38(4.50) | 6.38(12.87) | 6.03(0.63) |
| | X | 14.40(6.39) | 13.92(4.74) | 23.53(3.98) | 7.11(4.56) | 2.25(21.42) | 8.22(10.00) | 8.12(3.28) | 30.11(13.88) | 8.98(5.27) |

| | | | | | | | | | | |
|---|---|---|---|---|---|---|---|---|---|---|
| | P | 2.75(13.72) | 3.56(13.25) | 5.67(6.01) | 0.83(15.79) | 0.62(9.63) | 2.02(10.20) | 5.62(16.56) | 2.97(2.68) | 5.64(3.39) |
| **physcion** | CR | 4.72(14.36) | 6.18(12.13) | 2.94(14.16) | 2.04(4.55) | 1.25(11.52) | 5.11(19.60) | 1.57(11.98) | 3.14(12.76) | 2.40(4.80) |
| | CT | 2.46(7.55) | 5.20(6.39) | 3.80(13.01) | 1.64(18.98) | 2.31(24.48) | 7.04(4.77) | 1.26(5.25) | 2.85(4.86) | 2.28(3.89) |
| | XAB | 9.03(7.29) | 6.44(11.80) | 14.53(15.96) | 2.10(12.86) | 3.33(18.90) | 4.80(11.51) | 14.82(3.87) | 16.84(12.97) | 1.97(3.68) |
| | PAB | 0.78(25.83) | 1.00(1.59) | 0.94(26.94) | 0.76(11.41) | 0.83(28.51) | 1.93(13.06) | 1.22(1.63) | 1.52(3.77) | 1.66(6.76) |
| | X | 8.14(10.63) | 6.61(8.18) | 17.21(1.56) | 3.86(9.08) | 1.27(24.62) | 3.38(8.78) | 1.85(3.35) | 17.83(9.79) | 2.62(4.43) |
| | P | 0.98(15.93) | 0.88(27.62) | 1.65(6.78) | 0.88(24.32) | 0.90(8.10) | 1.02(8.72) | 1.26(9.19) | 1.11(5.31) | 1.48(8.95) |

Data in the table is an average of triplicate, and the numbers in parentheses/brackets are the RSD values in % of triplicate; * represent undetected.
